# The Hydrothermal-Assisted Approach Improves the Photocatalytic and Energy Storage Performance of Novel CuSe-TiO_2_-GO Composite

**DOI:** 10.3390/nano14131136

**Published:** 2024-07-01

**Authors:** Afaq Ullah Khan, Kamran Tahir, Muhammad Zia Ullah Shah, Hissah Saedoon Albaqawi, Zainab M. Almarhoon, Abdulaziz A. Alanazi, Nora Awad Alkudaisi, Talal M. Althagafi, Nacer Badi, Magdi E. A. Zaki

**Affiliations:** 1 School of Chemistry and Chemical Engineering, Jiangsu University, 301 Xuefu Road, Zhenjiang 212013, China; 2 Institute of Chemical Sciences, Gomal University, Dera Ismail Khan 24551, Khyber Pakhtunkhwa, Pakistan; 3 Faculty of Materials Science and Engineering, Kunming University of Science and Technology, Kunming 650093, China; 4 Department of Physics, College of Science, University of Ha’il, Ha’il P.O. Box 2440, Saudi Arabia; 5 Chemistry Department, College of Science, King Saud University, P.O. Box 2455, Riyadh 11451, Saudi Arabia; 6 Department of Chemistry, College of Science and Humanities in Al-Kharj, Prince Sattam bin Abdulaziz University, Al-Kharj 11942, Saudi Arabia; 7 Department of Physics, College of Science, Imam Abdulrahman Bin Faisal University, P.O. Box 1982, Dammam 31441, Saudi Arabia; 8 Department of Physics, College of Science, Taif University, Taif 21944, Saudi Arabia; 9 Thermal Management and Sustainability Research Laboratory, Department of Physics, Faculty of Science, University of Tabuk, Tabuk 71491, Saudi Arabia; 10 Department of Chemistry, College of Science, Imam Mohammad Ibn Saud Islamic University, Riyadh 11623, Saudi Arabia

**Keywords:** CuSe-TiO_2_-GO composites, methylene blue, photocatalytic properties, supercapacitor, electrode material

## Abstract

This study reports a novel CuSe-TiO_2_-GO composite, synthesized by a facile hydrothermal method at a controlled temperature, and investigates its electrochemical performance for supercapacitors (SCs) and photocatalytic behavior for degrading methylene blue (MB) dye. The compositional phase structure and chemical bond interaction were thoroughly investigated. The as-fabricated pristine, binary, and ternary composites underwent comprehensive characterization employing spectroscopic techniques and electrochemical analysis. Compared with pure and binary compounds (CuSe, TiO_2_, and binary CuSe-TiO_2_ composites), the ternary CuSe-TiO_2_-GO composites demonstrated a high degradation efficiency while degrading MB in less than just 80 min (240 min, 100 min, and 140 min, respectively). The photocatalytic activity of the ternary CuSe-TiO_2_-GO composites is enhanced due to the highly positive conduction band of CuSe, leading to the quick excitation of electrons to the conduction band of CuSe. Subsequently, graphene oxide (GO) left holes on the photocatalyst surface for MB, as GO assisted the photoexcited electron–hole pairs, resulting in enhanced photocatalytic performance. The CuSe-TiO_2_-GO electrode for the supercapacitor indicates a 310.6 F/g and 135.2 F/g capacitance when the discharge current upsurges from 1 to 12 A/g. The good photocatalytic and energy storage performance is due to the smaller charge transfer resistance, which promotes efficient separation of electron–hole pairs.

## 1. Introduction

Water is one of the primary requirements for a living organism. With the development of industries, water pollution has become a serious stumbling block due to industrial wastage in the water bodies [1,2,3,4]. Removing heavy metals, contaminations, drugs, pesticides, and organic dyes is highly necessary for water shacks before discharging. Due to their highly stable nature and complex structure, the organic dyes are purely degradable [5,6]. There are various methods employed for wastewater treatment, such as ion exchange, sedimentation, adsorption, etc., owing to their pros and cons, including expert labor to handle the process, high expense, and requiring highly time-consuming complex environmental conditions to operate [7,8,9]. The main flaw lies in that these conventional methods cannot separate organic dye from wastewater but instead convert it into another phase, which causes more danger to the environment. In the modern research society, photocatalysis is straightforward to handle, economical, safe, environmentally ambiguous, and a sustainable approach for converting organic pollutants into simplistic components [10,11]. Even though selenium is hazardous in tiny quantities, it has been shown to be an antagonist for other metal toxins such as cadmium, arsenic, mercury, and thallium. The importance of selenium in human nutrition and its different medical applications has generated contentious debate over the past 20 years. Selenium with copper has demonstrated remarkable photo activity for organic pollutant photodegradation, as reported in previous studies [12,13]. Additionally, TiO_2_ has been extensively investigated among other photocatalysis because it has many benefits such as non-toxicity for the environment, high separation rate of photogenerated carriers, contribution to redox activities, and so on [14,15,16,17]. The bandgap of TiO_2_ constrains its absorption to a small fraction of the solar spectrum. Therefore, doping with transition metals has been utilized as a technique to enhance light absorption within the visible region [18,19].

Due to a tiny bandgap, Cu-based materials (such as CuO, Cu_2_O, and CuSe) demonstrated remarkable photocatalytic activities. Among the copper chalcogenides listed, copper selenide has received a lot of interest due to its exceptional photochemical and optical capabilities. The CuSe nanocrystals that were produced had different shapes and structures depending on the precursors used and the reaction conditions. In particular, pure CuSe in the hexagonal phase is stable at room temperature and has been demonstrated to be an ideal precursor for the synthesis of CuInSe_2_-based nanostructures. The nanostructured materials based on CuInxGa1xSe2 have the most promise for use in solar energy generation [20,21]. The electro-optical properties, defect removal, particle size, and specific surface area of the photocatalyst have been discovered to be highly dependent on the photocatalytic activity [22]. The copper selenide quantum dots utilized in this study were created using a technique described in the literature [23].

Graphene is a two-dimensional material with unique electron promoter capabilities and outstanding electrochemical properties. Surface functioning is crucial for the attachment of CuSe nanoparticles to GO nanosheets. The GO has strong oxygen functional groups, including carboxyl, epoxy, and hydroxyl, demonstrating a high chemical reactivity and hydrophilicity [24,25]. Additionally, graphene has a two-dimensional layer structure, a greater surface area, chemical stability, and electrical conductivity, which leads to enhanced semiconductor photocatalytic efficacy as an extra agent. The combination of CuSe and GO (CuSe/GO composite) reduces electron–hole pair recombination, leading to an increase in photocatalytic activity (PCA) of semiconductor materials. This is because GO has a superior capacity to conduct electrons [26]. Therefore, to enhance the efficacy of the GO and TiO_2_ system, the incorporation of CuSe has gained considerable attention [27,28]. This integration shows considerable potential in the domain of selenide-based electrodes for electrochemical energy application, attributed to their remarkable features such as high conductivity, multiple metallic states, elevated theoretical capacity, and superior energy storage performance in aqueous electrolytes.

In this work, the optical properties of the CuSe, TiO_2_, binary CuSe-TiO_2_ composites, and ternary CuSe-TiO_2_-GO composites were presented, and their photocatalytic activities for the photodegradation of MB were carefully evaluated. When compared to the ternary CuSe-TiO_2_/GO composite, the optical investigation showed that the emission band in CuSe-TiO_2_-GO exhibited a significant blue shift. The CuSe-TiO_2_-GO and its composites (CuSe-TiO_2_ and CuSe-TiO_2_-GO composite) have bandgaps that are around 2.5 eV and 3.0 eV. The results demonstrate the photodegradation–adsorption advantages of CuSe-TiO_2_-GO composite, making it a possible new photocatalyst to decrease a variety of organic pollutants.

## 2. Sample Preparation

### 2.1. Materials

Elemental selenium (Se), Ofloxacin (98%), copper chloride (CuCl_2_·H_2_O), titanium isopropoxide (C_12_H_28_O_4_ Ti, 97%), CuCl_2_ (97.5%), Ofloxacin (98%), (hydrogen peroxide (H_2_O_2_), potassium permanganate (KMnO_4_), phosphoric acid (H_3_PO_4_), sulphuric acid (H_2_SO_4_), Sodium hydroxide (NaOH, 97%), acetic acid (CH_3_COOH, 98%), isopropyl alcohol (C_3_H_8_O, 98%), and absolute ethanol were purchased from Sinopharm Chemical Reagent Co., Shanghai, China. All chemicals used were of analytical grade and employed without further purification.

### 2.2. CuSe Preparation

To prepare a selenium alkaline aqueous solution, 3.948 g of elemental Se was poured into a beaker containing 12 M NaOH solution, and the mixture was stirred. Full elemental selenium caused a rapid color shift to orange–red, and the CuCl_2_ solution became a Cu^2+^ solution. Cu^2+^ was mixed with DI water and immediately stirred (and centrifuged) to remove any remaining alkaline solution. After drying the resulting product at 60 °C for 12 h, the CuSe powder was obtained.

### 2.3. TiO_2_ Preparation

Sodium hydroxide was used as the mineralizer, and analytical-grade titanium tetrachloride was used as the source material. After combining one molar TTIP stoichiometric ratio with 50 mL of distilled water, a titanium aqueous solution was obtained. A white colloidal sol is achieved by adding 2–3 mol of NaOH to a solution and stirring for a few minutes. Using distilled water, the final volume was adjusted to 90 mL. To avoid contamination, 90 mL of the solution was transferred to a 100 mL Teflon-lined autoclave vessel. To create TiO_2_ nanoparticles, the sealed container was heated for 12 h at 240 °C, and the residue that formed was then dried for 2 h at 450 °C.

### 2.4. Fabrication of GO

The modified Hummer’s approach was used for the synthesis of GO sheets. Furthermore, 10 mL of phosphoric acid and 90 mL of sulfuric acid were mixed and stirred for 10 min. After another 30 min of continuous stirring, 0.75 g of graphite powder was added to the mixture. Next, potassium permanganate was added dropwise. After that, the mixture was stirred for around 10 h. Then, 2.5 mL of hydrogen peroxide was added and cooled down. Finally, 15 mL of hydrochloric acid was added to the mixture above and allowed to sit for 24 h. After a thorough deionized water wash and centrifugation, the product was baked at 90 °C to dry.

### 2.5. Preparation of CuSe-TiO_2_-GO Ternary Composites

CuSe-TiO_2_ composites were prepared by changing the weight percent ratios of TiO_2_ sheets and CuSe nanoparticles. Briefly, 25% weight ratios of TiO_2_ (relative to CuSe) and CuSe nanoparticles were dispersed in 40 mL methanol via sonication. This mixture was stirred for around 30 min and carefully washed with DI water and ethanol. The product was finally dried at 60 °C; after that, the same procedure was followed for CuSe-TiO_2_-GO nanocomposites, with 50% weight ratios of CuSe-TiO_2_ (relative to GO).

### 2.6. Characterization

An X-ray Diffractometer was used to analyze the structural investigations of the produced powders (XRD, Thermo fisher scientific made, ARL Equinox 3000, MA, USA). The vibrational modes of the materials that were adjusted for were examined through the utilization of Raman spectroscopy, employing the Thermo Scientific, Waltham, MA, USA). The morphologies and elemental compositions of the produced samples were analyzed using field emission scanning electron microscopy (Hitachi S-4800 FE-SEM), in conjunction with energy-dispersive X-ray spectroscopy (SEM-EDX), manufactured in Tokyo, Japan. An electrochemical workstation (Interface 1010E, Gamry instruments, Warminster, PA, USA) was used to examine the electrochemical efficacy of both pure and composite samples. All electrochemical experiments were conducted in a 3 M KOH electrolyte, with pure and composite samples serving as working electrodes, a platinum (Pt) wire serving as a counter electrode, and Ag/AgCl working as a reference electrode. In order to evaluate the electrochemical characteristics, experiments utilizing a typical three-electrode setup were carried out. These tests included cyclic voltammetry (CV), electrochemical impedance spectroscopy (EIS), and galvanostatic charge–discharge (GCD).

### 2.7. Photocatalytic Experiments

The photocatalytic activity of CuSe, TiO_2,_ and GO and their composite was evaluated by decolorizing a 50 mg/L solution of MB. For each measurement, 100 mL of MB solution was taken in a 200 mL glass beaker. In the MB solution, a constant amount (0.02 g) of the synthesized photocatalyst powders (CuSe), (TiO_2_), (CuSe, TiO_2_), (CuSe, TiO_2_-GO) was poured. The same amount of CuSe, TiO_2_-GO (0.02 g), was used for all compositions to evaluate the degradation reaction rate. A Xenon lamp of 400 watts was used to examine the samples. First, an adsorption–desorption equilibrium between the MB and the synthesized powders was achieved by keeping the solution in the dark while stirring for 30 min. After reaching balance, the solutions were illuminated for 20 min with a xenon light while being continuously stirred. A UV–vis spectrophotometer was used to record the decolonization of MB solution at its greatest absorption peak (600 nm) (U-2900, spectrophotometer, Hitachi, Tokyo, Japan). Using Lambert–Beer’s law for the absorption data, the concentration of remaining MB in the solution was determined as a function of illumination time.

## 3. Results and Discussion

**Figure 1:**
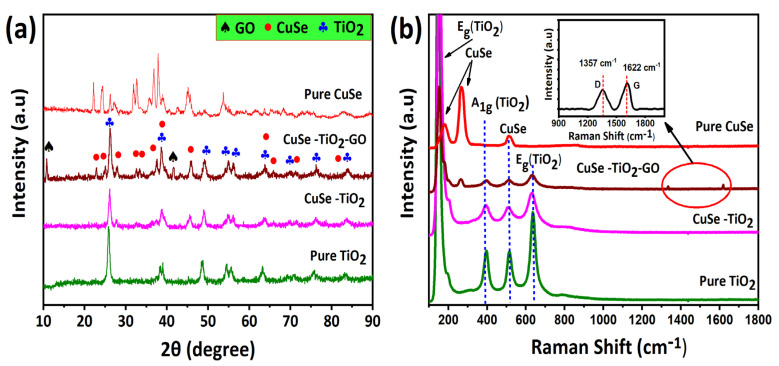
Phase and crystal structure analysis: (**a**) XRD pattern, (**b**) Raman spectra of CuSe, CuSe-TiO_2_, and CuSe-TiO_2_-GO composites.

The sample’s crystallinity, phase structure, and preparation were confirmed first via X-ray diffraction (XRD) characterization, as presented in Figure 1. The XRD pattern of the samples is given in Figure 1a. The crystal phase of TiO_2_, known as tetragonal anatase, may be identified by its JCPDS No: 01-084-1285. This phase exhibits distinct 2θ values at various angles, including 25.30°, 38.5°, 48.03°, 53.8°, 55°, 62.69°, 68.76°, 70.2°, 75.05°, and 82.76°. These angles correspond to certain crystallographic planes, including (101), (112), (200), (1050, (211), (204), (116), (220), (215), and (224). Additionally, the diffraction pattern of CuSe exhibited distinct peaks at angles of 26.2°, 31.7°, 32.6°, 45.5°, 53.3°, 57°, and 65.2°. These peaks corresponded to the miller indices of (100), (001), (120), (110), (200), (111), (002), and (208), respectively. This crystal structure was determined to match with the hexagonal crystal structure of CuSe, which is consistent with the standard of CuSe as per JCPDS#00-027-0185. Additionally, some other peaks related to CuSe_2_ diffraction peaks at 35.8°, 38.4°, 61.4°, and 62.5° miller indices (200), (210), (311), and (212) with JCPDS#01-074-0280 were also detected. Similarly, the XRD pattern revealed the presence of GO peaks at a 2θ value of 10.4°, which corresponds to the (001) plane, and at 43.4°, corresponding to the (111) plane. The CuSe-TiO_2_-GO composite showed no additional peaks that stipulated the high crystallinity and purity of the samples. The only difference was that the intensity was decreased, demonstrating the low crystallinity of the ternary composite. Using the Scherrer equation, the crystal size of the synthesized materials was calculated. The equation is as follows:

(1) $$D=\frac{K\lambda }{\beta cos\theta }$$

**Figure 2:**
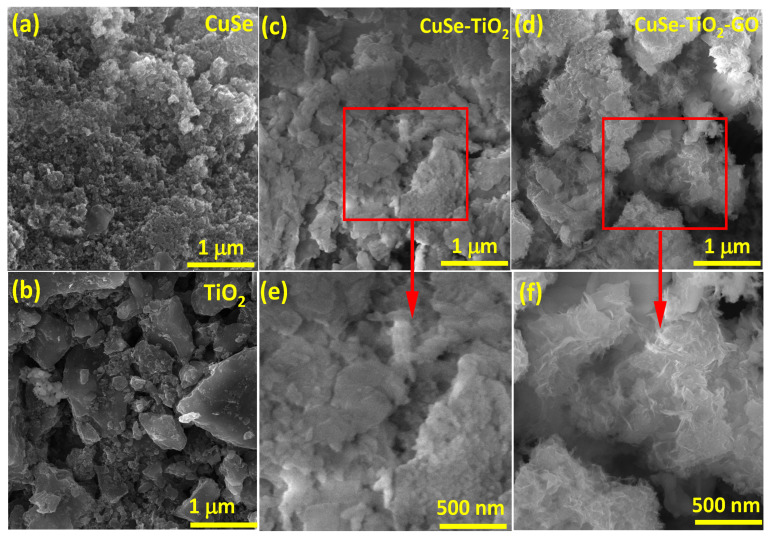
SEM images of (**a**) CuSe, (**b**) TiO_2_, (**c**,**e**) CuSe-TiO_2_, (**d**,**f**) CuSe-TiO_2_-GO composites.

In the above equation, the crystalline size of the material is represented by D, *K* is the Scherrer constant with a value of 0.94 for spherical crystallite size with cubic symmetry, the wavelength of the X-ray is represented by λ (a constant value equal to Cu κα = 0.154 nm), ‘β’ is full width at half maximum (FWHM), and ‘θ’ is the diffraction angle [29]. The average crystalline size was calculated for CuSe, TiO_2_, and CuSe-TiO_2_-GO composite, as tabulated in Table 1. Compared to the binary CuSe-TiO_2_ composite, the ternary CuSe-TiO_2_-GO composite displayed a smaller crystalline size among all samples. Raman spectroscopy provides detailed information about chemical structures and molecular interaction. The Raman spectra of the prepared samples are given in Figure 2b. The low-temperature TiO_2_ indicates four distinctive Raman shifts, 150 cm^−1^ (Eg), 410 cm^−1^ (A_1g_), 520 cm^−1^ (B_1g_), and 620 cm^−1^ (E_g_). Furthermore, due to the Cu-Se connection, the pure CuSe sample revealed three vibrational modes at 209.3 cm^−1^ (A_1g_), 256.4 cm^−1^, and 492.7 cm^−1^ (E_g_) that may be ascribed to the stretching vibrational mode (A_1g_) [30,31]. Noteworthy, the CuSe-TiO_2_ composite revealed the combined peaks from CuSe and TiO_2_ compounds, describing the development of the CuSe-TiO_2_ composite. Similar Raman vibrational modes were declared when moving toward the ternary CuSe-TiO_2_-GO composite. As shown in the inset of Figure 1b, two new peaks appear at 1357 cm^−1^ owing to the D and G bands around 1622 cm^−1^. A low Raman intensity was also noticed in the ternary composite, according to the XRD investigation.

The surface morphology and overall appearance of the pure, binary, and ternary composites can be analyzed by SEM. The CuSe sample shows a minute size of nanoparticles (NPs) with aggregation, which makes a honey-bee nest-like architecture from the overall surface view (see Figure 2a). Meanwhile, the pure TiO_2_ sample indicates merged surfaces and irregular morphology with aggregation and some small particles scattered on the surface (see Figure 2b). The large agglomeration of the particles may be due to the Wander Waals forces, which combined the particles [32,33,34]. The CuSe-TiO_2_ and CuSe-TiO_2_-GO composites displayed a highly agglomerated surface appearance (see Figure 2c–f. The influence of the Wander Waals forces increases, and the morphology of the binary CuSe-TiO_2_ and ternary CuSe-TiO_2_-GO composites becomes complex and dense, forming more rough surfaces, which contributes to the enhancement of the surface area. The GO is attached to the overall texture, causing a crumpling and aggregated flakes with many layers. The introduction of GO does not make any possible changes in the surface morphology of ternary CuSe-TiO_2_-GO composites, indicating the good structural feature and combination of the composite material.

**Figure 3:**
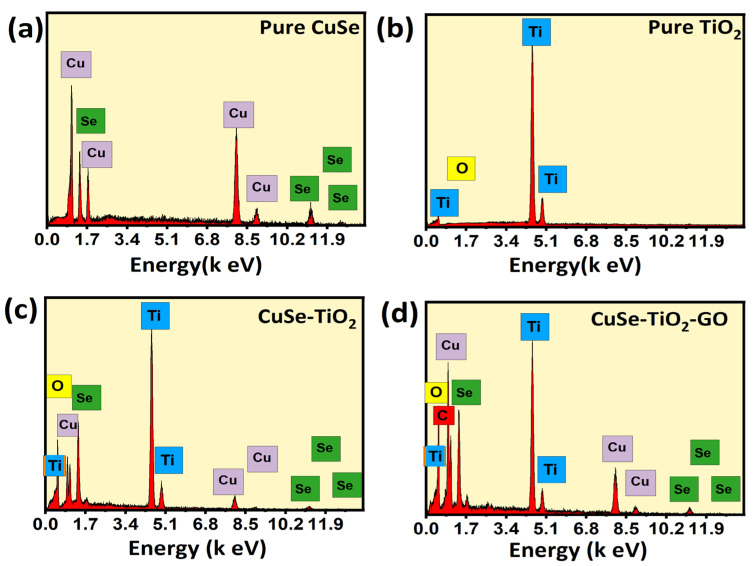
EDX spectrum of (**a**) CuSe, (**b**) TiO_2_, (**c**) CuSe-TiO_2_, (**d**) CuSe-TiO_2_-GO composites.

**Figure 4:**
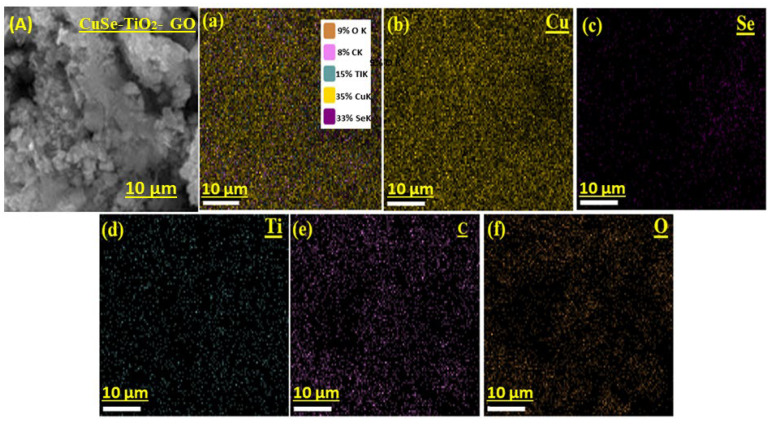
(**a**–**f**) The corresponding elemental mapping of CuSe-TiO_2_-GO composites.

The EDX spectra confirmed the phase purity and elemental compositional analysis of the pure, binary, and ternary composite materials, as revealed in Figure 3. As indicated in Figure 3a,b, the pure samples CuSe and TiO_2_ manifest the presence of only Cu, Se, Ti, and O-containing elements without any foreign elements in their structure, implying high purity and good formation of the desired samples individually. Similarly, the spectra of the binary CuSe-TiO_2_ composite validate the persistence of the corresponding elements such as Cu, Se, Ti, and O elements, again confirming the high purity of the binary composite, as shown in Figure 3c. Furthermore, the EDX spectra of the ternary CuSe-TiO_2_-GO composite (see Figure 3d) showed CuSe and TiO_2_-containing elements, with an additional C peak also detected, signifying the introduction of GO in the composite material. Thus, the EDX results also support the findings of the XRD and SEM studies. Moreover, elemental mapping is also attached with EDX spectra, as displayed in Figure 4a–f.

**Figure 5:**
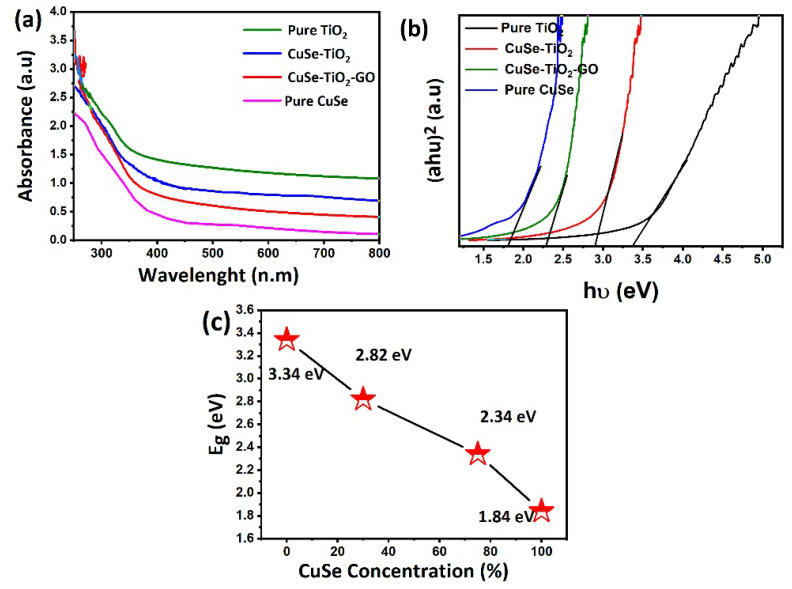
Optical properties of CuSe, CuSe-TiO_2_, and CuSe-TiO_2_-GO composites: (**a**) UV-Vis spectra, (**b**) bandgap plot, (**c**) concentration of CuSe vs. bandgap plot.

The optical properties of CuSe, TiO_2_, binary CuSe-TiO_2_ composites, and the ternary CuSe-TiO_2_-GO composite were analyzed using UV-Vis absorption spectroscopy. The UV results of the samples are illustrated in Figure 5. It was revealed that all samples showed a maximum absorption below 300 nm wavelength. The pure CuSe and ternary CuSe-TiO_2_-GO composite indicate a slightly longer absorption edge than pure TiO_2_ and binary CuSe-TiO_2_, leading to low bandgap energy. This can be confirmed by optical bandgap analysis, as demonstrated in Figure 5b. Likewise, the smaller bandgap energy (2.34 eV) of the ternary CuSe-TiO_2_-GO composite revealed the maximum absorption in the UV-Vis region, as indicated in Figure 5c. It demonstrates that compositing is one of the effective strategies to reduce the bandgap energy of excellent photocatalytic materials (e.g., here TiO_2_) and further promotes enhanced photocatalytic performance.

**Figure 6:**
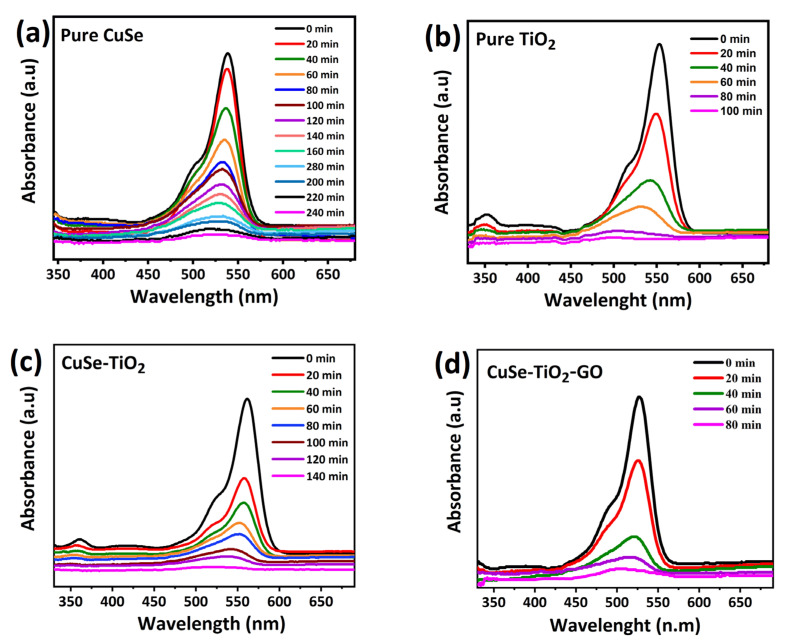
UV-Vis absorption spectra of (**a**) CuSe, (**b**) TiO_2_, (**c**) CuSe-TiO_2_, (**d**) CuSe-TiO_2_-GO composites.

**Figure 7:**
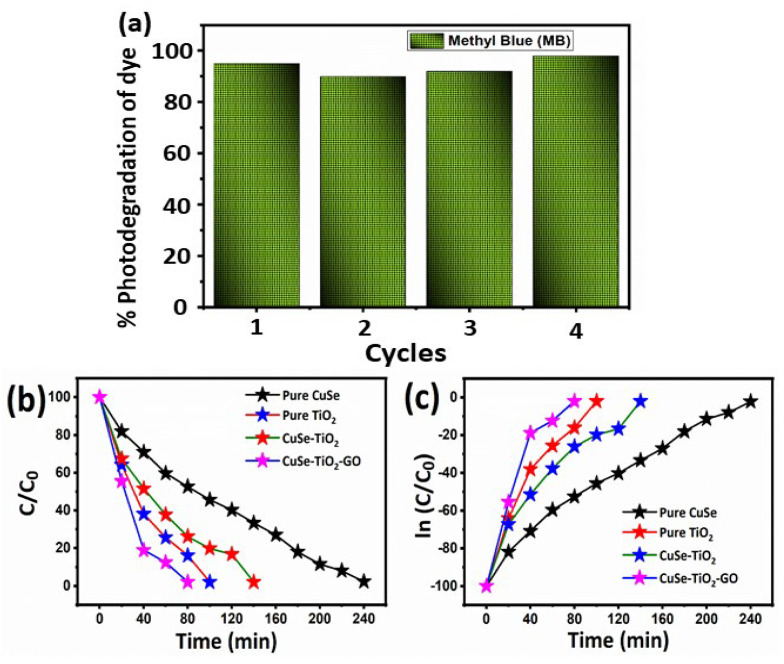
(**a**) Recyclability study, (**b**) photodegradation percentage (%) of CuSe, TiO_2_, CuSe-TiO_2_, and ternary CuSe-TiO_2_-GO composites under visible light irradiation, and (**c**) UV-vis time vs. log plot of their composites.

The first step in photocatalytic dye degradation is the absorption of the dye on the surface of the catalyst. Initially, a 50 mg/L starting concentration and the strength of the MB absorption peak were used to assess how much MB was absorbed by the photocatalyst (541 nm). The results of an impressive investigation into the photocatalytic activities against the MB dye for the CuSe, TiO_2_, CuSe-TiO_2_, and ternary CuSe-TiO_2_-GO samples are displayed in Figure 6 and Figure 7. In this investigation, MB is used under the conditions that, as previously described, provide the relevant samples with the greatest photocatalytic performance. The Xe lamp was also started to research photocatalytic activity when exposed to light. Pure samples, such as TiO_2_ and CuSe, have CuSe that is more responsible for the catalytic performance (Figure 6a,b), and their composites displayed in Figure 6c,d show the peak intensity decreases with increasing irradiation time. Following that, when compared to the composites (CuSe-TiO_2_ and CuSe-TiO_2_-GO), the ternary CuSe-TiO_2_-GO composites exhibit superior catalytic performance, as they take less time than their counterparts to clear the MB dye solution. The CuSe-TiO_2_ composite sample had the highest adsorption %, according to the results. The high adsorption efficiency of CuSe-TiO_2_ composites is related to their structure, which includes a lack of crystalline order and the presence of many gaps and fissures in the material. To further verify the MB enhanced performance, the stability test was run a total of four times, as shown in Figure 7a. A similar lack of change in the photocatalytic degradation of CuSe-TiO_2_-GO was found, and the rate of degradation is approximately 94%, indicating the reusability of the catalyst.

In order to reach the adsorption–desorption equilibrium, each solution was pre-treated in the dark for one hour before being irradiated with UV light. The remaining MB concentrations were calculated by analyzing UV-Vis spectra at the central absorption peak, which was centered at 541 nm. The photocatalytic activity of TiO_2_ and GO-based catalysts was investigated using MB. As can be seen in Figure 7b,c, the photodegradation rate is presented as the ratio of the initial dye concentration of MB (C_0_) to the final concentration (C/C_0_), where C represents the MB concentration at time t. Interestingly, ternary CuSe-TiO_2_-GO had more photocatalytic activity than CuSe-TiO_2_. Following that, the ternary CuSe-TiO_2_-GO composites degrade MB up to 98% in 80 min, whereas the CuSe-TiO_2_, CuSe (240 min), TiO_2_ (100 min), and CuSe-TiO_2_ degrade just 98% in 140 min. Table 2 presents the results of investigations that compare the stated photocatalytic efficacy of a synthesized composite with reported photocatalysts. As shown in this work, CuSe-TiO_2_ has superior photocatalytic activity when compared to CuSe, indicating that TiO_2_ and GO-based nanomaterials may be outstanding photocatalysts for real-world applications.

The mechanistic study reveals that the efficacy of photocatalytic degradation of dye depends on various factors, i.e., light harvesting efficiency and photogenerated electron–hole charge reactions with substrate molecules. Basically, the photodegradation of dye occurs upon interaction with UV light and the photocatalyst. The photon possessing energy equal to or exceeding the bandgap of the catalyst promotes the excitation of electrons from the valence band (VB) to the conduction band (CB), generating positive holes (h+) in the VB. These h ions react with the water to generate hydroxyl radicals (·OH), while the CB electrons interact with oxygen, leading to the formation of superoxide anion (O_2_·^−^) [39]. These radicals exhibit high reactivity and efficiently decompose dye molecules into simpler compounds such as H_2_O and CO_2_. In pristine TiO_2_, a substantial portion of the separated electron–hole pairs recombine, diminishing photocatalytic activity. However, in CuSe-TiO_2_ and CuSe- TiO_2_-GO composites, coupled CuSe and GO capture electrons from the TiO_2_ valence band, resulting in a decreased recombination rate of electron–hole pairs. Consequently, the enhanced efficacy renders the composite with more potent photocatalysis than pure TiO_2_.

**Figure 8:**
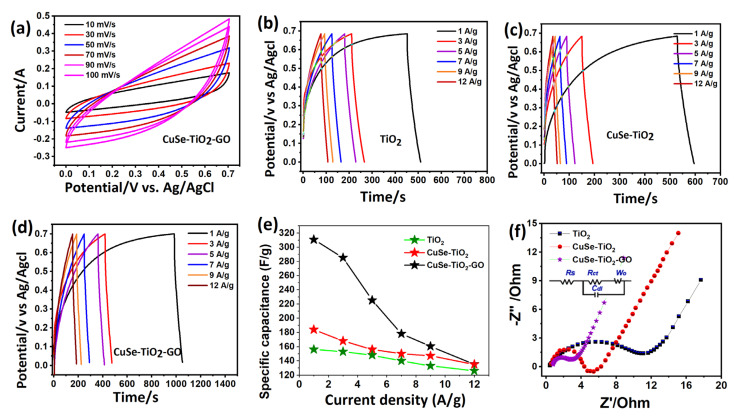
Electrochemical characterization of the CuSe-TiO_2_-GO composite, (**a**) CV curves of CuSe-TiO_2_-GO composite, GCD profile of (**b**) TiO_2_, (**c**) CuSe-TiO_2_, (**d**) CuSe-TiO_2_-GO composite, (**e**) capacitance plot, and (**f**) impedance plot.

The best ternary Cu-Se-TiO2-GO composites, inspired by their excellent photocatalytic performance, are the ideal option for electrochemical charge storage properties for supercapacitor applications, as demonstrated in Figure 8. The electrochemical properties were measured using CV and GCD tests at different current densities in a fixed potential range. CV curves were taken in an alkaline electrolyte solution containing 3M KOH in a potential region of 0.0 to 0.7 V for different scan speeds, as stipulated in Figure 8a. A combined charge storage behavior was observed in the CV curves with a quasi-rectangular shape due to GO. The good capacitive signature was attained by upsurging the enclosed loop area when the scanning speeds increased from low to higher values. This trend specifies the rapid diffusion of ions during the electrochemical process, which can be further confirmed from charge–discharge analysis, as shown in Figure 8b–d. Concurrently, the TiO_2_, CuSe-TiO_2_, and CuSe-TiO_2_-GO nanocomposite electrodes exhibited nonlinearity, indicative of a battery-type discharge–charge profile. An initial notable reduction in iR was observed in all GCD curves, attributed to the heightened internal resistance during rapid discharge–charge processes. Across various discharge current responses for all electrodes (Figure 8b–d), GCD curves demonstrated consistency, highlighting commendable energy storage and rate capabilities. Remarkably, the CuSe-TiO_2_-GO composite electrodes showcased superior energy storage capacities, as evidenced by prolonged discharge times compared to their counterparts (see Figure 7d), and the related comparative plot is shown in Figure 8e. The decline in capacitance was usually observed from low to high current rates due to less ion–mass diffusion. Only the outer surface is active for charge accumulations [40,41,42], as depicted in Figure 8e. The charge–discharge profile of the ternary CuSe-TiO_2_-GO composite reflects a similar potential window at alternative discharge currents, indicating the stable morphology due to multi-component synergy in the final ternary compound. Discharge capacities were determined using Equation (2), and their values for all electrodes, regardless of their respective discharge currents, are delineated in Table 3.

(2) $$C=\frac{it}{mV}$$

where i/m denotes the current and mass loading on Ni foam, t is the discharge time, and V is the potential difference of the CD or CV voltammogram.

The impedance plot further confirms the low charge transfer resistance of the ternary CuSe-TiO_2_-GO composite, supporting the fast charge transport kinetic due to the high conductivity offered by GO in the composite electrode. Interestingly, the CuSe-TiO_2_-GO composite electrode displayed a smaller semicircle size and a sharper vertical line compared to TiO_2_ and CuSe-TiO_2_ electrodes, implying significant capacitive behavior. The Rs values of TiO_2_, CuSe-TiO_2_, and CuSe-TiO_2_-GO composite electrodes are 0.2 Ω, 0.2 Ω, and 0.1 Ω, respectively, with their corresponding Rct values being 11.7, 5.8, and 2.9 Ω, as displayed in Figure 8f. Specifically, the CuSe-TiO_2_-GO composite electrode demonstrates the smallest semicircle (indicative of a low Rct value of 2.9 Ω) and the shortest point on the real axis (attributed to a small Rs of 0.1 Ω) compared to TiO_2_ and CuSe-TiO_2_ electrodes.

## 4. Conclusions

In the concluding remarks, the key findings of this study are presented as follows:

- The synergy between different nanomaterials in the composite and the highly conductive skeleton promotes the effective interaction of the GO with the CuSe-TiO_2_ composite;
- The ternary CuSe-TiO_2_-GO composites showed enhanced photocatalytic properties due to the good optical properties of CuSe and the high efficiency of GO to upgrade the photoexcited electron–hole pairs for the catalyst under visible light irradiation;
- The composites with the relative ratios of CuSe, TiO_2_, and GO (CuSe-TiO_2_ and CuSe-TiO_2_-GO) outperformed their pure CuSe and GO counterparts in photocatalytic activity. The ternary CuSe-TiO_2_-GO composite demonstrated remarkable performance in this study;
- For supercapacitor application, the ternary CuSe-TiO_2_-GO composite exhibits enhanced energy storage performance, including capacitance, low charge transfer resistance, and rapid charge transport kinetics facilitated by its high conductive matrix, thereby achieving superior performance.

This work suggests the optimistic route to develop other selenide-based GO composites to utilize photocatalytic activities effectively.

## Figures and Tables

**Table 1:** Crystallite size of the CuSe, TiO_2_, CuSe-TiO_2_, and CuSe-TiO_2_-GO composites.

Sample Name	Average Crystallite Sizes (nm)
**CuSe**	23.42
**TiO_2_**	17.4
**CuSe-TiO_2_**	21.52
**CuSe-TiO_2_-GO**	14.18

**Table 2:** Comparative study of the photodegradation of MB by the related photocatalyst.

Catalyst	Dye	Light Source	%Dye Degradation	Time (min)	References
Ni-Cr/TiO_2_	MB	Sunlight	95.6%	90	[35]
CeO_2_-NPs/GO/PAM	MB	UV	90%	90	[36]
ZnO@OFE	MB	sunlight	75%	180	[1]
TiO_2_/CA	MB	UV	93.86%	80	[37]
GO/ZnO	MB	300 w Xe lamp	86.86%	100	[38]
CuSe-TiO_2_-GO	MB	400 w Xe lamp	98%	**80**	**This work**

**Table 3:** The specific capacitance of TiO_2_, CuSe-TiO_2_, and CuSe-TiO_2_-GO composite electrodes at different discharge current rates.

Current Density (A g^−1^)	TiO_2_ (F g^−1^)	CuSe-TiO_2_ (F g^−1^)	CuSe-TiO_2_-GO (F g^−1^)
**1**	156	184	310.6
**3**	153	168	285.3
**5**	148	156	225.1
**7**	140	150	178
**9**	133	147	160.5
**12**	126	135	135.2

## Data Availability

Data are contained within the article.
